# Reactive lymphoid hyperplasia of the appendix in children: a descriptive analysis of accompanying neural and stromal features

**DOI:** 10.3389/fped.2026.1749724

**Published:** 2026-05-01

**Authors:** Neslihan Gulcin, Ilkay Tosun, Ceyhan Sahin

**Affiliations:** 1 Department of Pediatric Surgery, University of Health Sciences, Umraniye Training and Research Hospital, Istanbul, Türkiye; 2 Department of Pathology, University of Health Sciences, Umraniye Training and Research Hospital, Istanbul, Türkiye

**Keywords:** neural proliferation, pediatric appendectomy, perineural fibrosis, reactive lymphoid hyperplasia, S-100 immunohistochemistry

## Abstract

**Background:**

Reactive lymphoid hyperplasia (RLH) is frequently identified in pediatric appendectomy specimens performed for suspected acute appendicitis. However, its associated neural and stromal alterations remain incompletely characterized.

**Methods:**

This retrospective descriptive study evaluated pediatric appendectomy specimens obtained between January 2019 and January 2024. RLH cases were identified based on histopathological criteria. Archived specimens were re-evaluated using hematoxylin and eosin staining, S-100 immunohistochemistry, and Masson's trichrome staining. Neural alterations were classified according to Höfler's criteria, and fibrosis was graded semiquantitatively.

**Results:**

Among 468 appendectomies, 100 cases fulfilled the criteria for RLH. The mean age was 11.34 ± 4.31 years, and 63% were male. Neural and stromal alterations, including S-100–positive nerve elements and varying degrees of fibrosis, were frequently observed. Höfler Type 1 and Type 3 patterns were the most common, and fibrosis was predominantly mild to moderate. Postoperative symptom resolution was observed in patients with available follow-up.

**Conclusion:**

RLH in children is commonly associated with reactive neural and stromal alterations. However, these findings should be interpreted as nonspecific reactive changes rather than disease-specific features. Further studies are needed to clarify their clinical significance.

## Highlights

• Reactive lymphoid hyperplasia (RLH) is frequently accompanied by reactive neural and stromal features in pediatric appendectomy specimens.
• These neural and fibrotic changes occur even in the absence of classical inflammatory findings.
• RLH should not be misinterpreted as a completely normal appendix in pathology practice.
• Ancillary stains such as S-100 and Masson's trichrome improve recognition of subtle neural and fibrotic alterations.
• Careful documentation of these patterns may assist clinicians in understanding non-inflammatory causes of appendicitis-like presentations.

## 1 Introduction

Acute appendicitis is one of the most common surgical emergencies in children, typically presenting with nonspecific symptoms such as abdominal pain, nausea, and vomiting. In a subset of pediatric patients undergoing appendectomy for suspected appendicitis, histopathological evaluation reveals reactive lymphoid hyperplasia (RLH) rather than acute inflammation (1, 2). RLH is characterized by prominent lymphoid follicular proliferation within the lamina propria or submucosa, with preserved mucosal architecture and absence of neutrophilic infiltration (3).

RLH represents a reactive condition that may clinically and radiologically mimic acute appendicitis but does not fulfill the histological criteria of inflammation (3, 4). As part of the gut-associated lymphoid tissue (GALT), the pediatric appendix is particularly prone to reactive lymphoid changes in response to various immunological or luminal stimuli, including viral infections and mesenteric lymphadenitis (5, 6). Therefore, RLH may reflect a physiological or adaptive response rather than a distinct pathological entity, and this distinction is important to avoid overinterpretation of common pediatric histological findings.

Neural and stromal alterations of the appendix, including nerve fiber proliferation, Schwann cell prominence, and perineural fibrosis, have been described in both inflammatory and non-inflammatory conditions (7, 8). Höfler categorized these changes under the concept of neuropathic appendicopathy (8, 9), although similar findings have been reported across a wide range of gastrointestinal disorders, suggesting that they are likely nonspecific and reactive in nature (9, 10). Neural proliferations associated with fibrous obliteration of the appendiceal lumen have also been documented independently of acute inflammation, further supporting the notion that these alterations are not disease-specific (10).

In clinical practice, appendectomy specimens lacking acute inflammation are often labeled as “negative appendectomy.” However, this terminology may oversimplify the underlying histopathological spectrum, as RLH and other reactive changes may still be present (4, 11). Although RLH is generally considered a benign finding, it should not necessarily be equated with a completely normal appendix (12, 13).

Given these considerations, further characterization of RLH and its associated histopathological features in children is warranted. Therefore, the aim of this study was to provide a descriptive evaluation of the clinical, laboratory, imaging, and histopathological findings of pediatric patients with RLH who underwent appendectomy for suspected acute appendicitis. In addition, neural and stromal alterations were documented using ancillary staining methods and classified according to Höfler's criteria (9). Importantly, this study is designed as a descriptive analysis and does not aim to establish causality or disease specificity.

## 2 Materials and methods

### 2.1 Ethics approval

This study was approved by the Ethics Committee of Umraniye Training and Research Hospital (Approval No. 115; April 24, 2024). All procedures were performed in accordance with the Declaration of Helsinki. As this was a retrospective review of anonymized pathology and clinical records, the requirement for informed consent was waived by the ethics committee.

### 2.2 Patient selection

This retrospective study included pediatric patients (<18 years) who underwent appendectomy for suspected acute appendicitis between January 2019 and January 2024 at a tertiary care hospital. Pathology archives were systematically reviewed, and cases were initially identified based on routine evaluation of hematoxylin and eosin (H&E)–stained slides.

Specimens without histological evidence of acute inflammation and those with equivocal or borderline histopathological findings were included for diagnostic reassessment. Final case classification was established following standardized histopathological review.

This stepwise selection and re-evaluation approach was implemented to improve diagnostic accuracy; however, it may have introduced a degree of selection bias by enriching the cohort with cases requiring additional histopathological assessment.

### 2.3 Study design and sample processing

The study was designed as a retrospective descriptive histopathological analysis of appendectomy specimens diagnosed with reactive lymphoid hyperplasia (RLH). Specimens with acute appendicitis were reviewed only to confirm diagnostic categorization and were not included as a comparison group, as no statistical comparisons were planned. Accordingly, this study was not designed to determine disease specificity or to perform intergroup analyses, but rather to provide a descriptive characterization of RLH.

For RLH cases, all available hematoxylin and eosin (H&E)–stained slides were retrieved from pathology archives and re-evaluated. All RLH cases underwent reassessment. When required, additional levels were obtained from paraffin-embedded tissue blocks. The appendix was not routinely submitted in its entirety; however, additional sampling had been performed in cases with atypical features or diagnostic uncertainty, as documented in pathology records.

Two ancillary stains were used for further morphological characterization:

1. S-100 immunohistochemistry to assess Schwann cell prominence, nerve fiber distribution, and features of neural proliferation.
2. Masson's trichrome stain to evaluate stromal and perineural fibrosis.

The use of additional sectioning and ancillary staining may have increased the detection of neural and stromal features compared with routine pathological evaluation and should be considered when interpreting the findings.

### 2.4 Classification of neuropathic features

Neuropathic alterations were categorized according to Höfler's classification system, which includes:

1. Type 1: mucosal variant
2. Type 2: axial neuroma
3. Type 3: submucosal neuromuscular proliferation
4. Type 4: perineural fibrosis

The degree of perineural or stromal fibrosis was semiquantitatively graded as mild, moderate, or severe based on staining distribution and intensity, according to previously published criteria (5, 9).

### 2.5 Histopathological definitions

Reactive lymphoid hyperplasia (RLH) was defined as prominent lymphoid follicular hyperplasia within the lamina propria or submucosa, with preserved crypt architecture and absence of neutrophilic infiltration, cryptitis, ulceration, or transmural inflammation.

Acute appendicitis was defined by transmural neutrophilic inflammation, mucosal ulceration, crypt destruction, and edema. These diagnostic criteria were used solely to validate case classification within the dataset and not for comparative analysis.

### 2.6 Clinical data collection

Demographic data (age and sex), surgical approach (laparoscopic or open), and gross pathological features (appendix length and diameter) were extracted from operative and pathology reports. Imaging parameters—including appendiceal diameter, echogenicity, free fluid, and lymphadenopathy on ultrasonography—were documented when available. Laboratory values, including white blood cell (WBC) count and C-reactive protein (CRP) levels, were collected from electronic medical records. Postoperative clinical information, including symptom resolution and length of hospital stay, was reviewed when accessible. As this was a retrospective study, the availability and completeness of clinical and imaging data were variable and dependent on existing medical records.

All data were compiled to provide a comprehensive descriptive profile of pediatric patients diagnosed with RLH. No attempt was made to establish causal relationships between histopathological findings and clinical presentation.

In our institution, the clinical management of patients with suspected acute appendicitis is based on standard pediatric surgical protocols. All patients undergo clinical evaluation, laboratory testing, and ultrasonographic assessment. Perioperative antibiotic therapy is routinely administered in patients with suspected appendicitis. A single preoperative dose of broad-spectrum antibiotics is given, followed by postoperative antibiotics when clinically indicated. The choice and duration of antibiotic therapy are determined according to institutional guidelines and intraoperative findings.

As this study is retrospective and descriptive in nature, detailed antibiotic regimens were not analyzed separately; however, standard management protocols were applied consistently across patients.

### 2.7 Statistical analysis

The study was designed as a retrospective descriptive observational analysis. In accordance with the study objectives, no statistical comparisons were performed between RLH cases and acute appendicitis, as the aim was not to conduct intergroup hypothesis testing or determine disease specificity, but to provide a descriptive characterization of RLH.

All analyses were conducted using IBM SPSS Statistics for Windows, version 30.0 (IBM Corp., Armonk, NY, USA). Continuous variables were summarized as mean ± standard deviation (SD) or median with interquartile range (IQR), depending on data distribution. Categorical variables were reported as frequencies (n) and percentages (%).

This analytical approach was selected to present the clinical, laboratory, imaging, and histopathological features of RLH in a descriptive manner, without inferring causality or implying associations beyond the available data.

## 3 Results

### 3.1 Patient characteristics

During the study period, a total of 468 pediatric appendectomies were performed. Among these, 108 specimens (23%) showed no histological evidence of acute inflammation. In addition, 32 cases with equivocal histopathological findings were included for re-evaluation, yielding a total of 140 cases.

Following standardized histopathological reassessment, 100 cases fulfilled the predefined criteria for reactive lymphoid hyperplasia (RLH) and constituted the final study cohort. The remaining 40 cases did not meet the criteria for RLH and were therefore excluded from further analysis.

The mean age of individuals diagnosed with RLH was 11.34 ± 4.31 years, and males constituted the majority (63%). The laparoscopic approach was the most commonly used surgical method. No complications were noted during or immediately following surgery.

Morphometric assessment of RLH specimens demonstrated a mean appendiceal length of 6.67 ± 1.57 cm and a mean appendiceal diameter of 0.71 ± 0.52 cm (median: 0.6 cm, IQR: 0.5–0.8).

Table 1 summarizes the demographic, clinical, laboratory, imaging, morphometric, and operative characteristics of patients diagnosed with RLH.

**Table 1:** Demographic, clinical, laboratory, imaging, morphometric, and operative characteristics of pediatric patients with reactive lymphoid hyperplasia (RLH) (*n* = 100).

Variable	Value
Age (years)	11.4 ± 4.3 (median 11.7, IQR: 2–17)
Sex (*n*, %)	
Male	63 (63%)
Female	37 (37%)
Symptoms (*n*, %)	
Abdominal pain	100 (100%)
Nausea/vomiting	54 (54%)
Fever	22 (22%)
Laboratory findings (*n*, %)	
WBC	>10,000: 41 (41%)
CRP	>5 mg/L: 28 (28%)
Ultrasonographic findings (*n*, %)	
Appendiceal diameter >7 mm	49 (49%)
Increased echogenicity	32 (32%)
Free fluid	18 (18%)
LAP	27 (27%)
CT performed, *n* (%)	11 (11%)
MRI performed, *n* (%)	0 (0)
Pathological examination	
Appendix length (cm)	6.67 ± 1.57
Appendiceal diameter (cm)	0.71 ± 0.52
Operative approach (*n*, %)	
Laparoscopic	88 (88%)
Open	12 (12%)

Age, sex, presenting symptoms, laboratory markers (WBC, CRP), ultrasonographic findings, use of cross-sectional imaging, surgical approach, appendiceal morphometry are summarized. The data represent only the descriptive characteristics of the RLH cases; no comparative analyses were performed.

### 3.2 Histopathological findings in RLH specimens

Upon reevaluation of the appendectomy specimens diagnosed with RLH, prominent lymphoid follicles were observed, along with preserved mucosal architecture and the absence of neutrophilic infiltration, cryptitis, ulceration, or transmural inflammation.

Table 2 summarizes the histopathological, neural, and stromal features of the cases diagnosed with RLH.

**Table 2:** Histopathological and stromal features of reactive lymphoid hyperplasia (RLH) of the appendix (*n* = 100).

Parameters	*n* (%)
Höfler Classification	
Type 1—Mucosal	46 (46.0)
Type 2—Axial neuroma	4 (4.0)
Type 3—Submucosal neuromuscular proliferation	30 (30.0)
Type 4—Perineural fibrosis (including fibrous obliteration)	14 (14.0)
Mixed (Type 1 + Type 3) patterns	6 (6.0)
Fibrosis Grade	
Mild	67 (67.0)
Moderate	25 (25.0)
Severe	8 (8.0)
Additional Histological Findings	
Lymphoid follicles prominent	100 (100)
Mucosal integrity preserved	100 (100)
Cryptitis/neutrophils	0 (0)
Ulceration	0 (0)
Transmural inflammation	0 (0)

Distribution of Höfler classification subtypes [Types 1–4 and Mixed (Type 1 + Type 3) patterns], semiquantitative fibrosis grading, and additional histological features, including lymphoid follicle prominence, preserved mucosal integrity, and absence of neutrophilic inflammation. The findings reflect features observed exclusively in RLH specimens.

**Figure 1:**
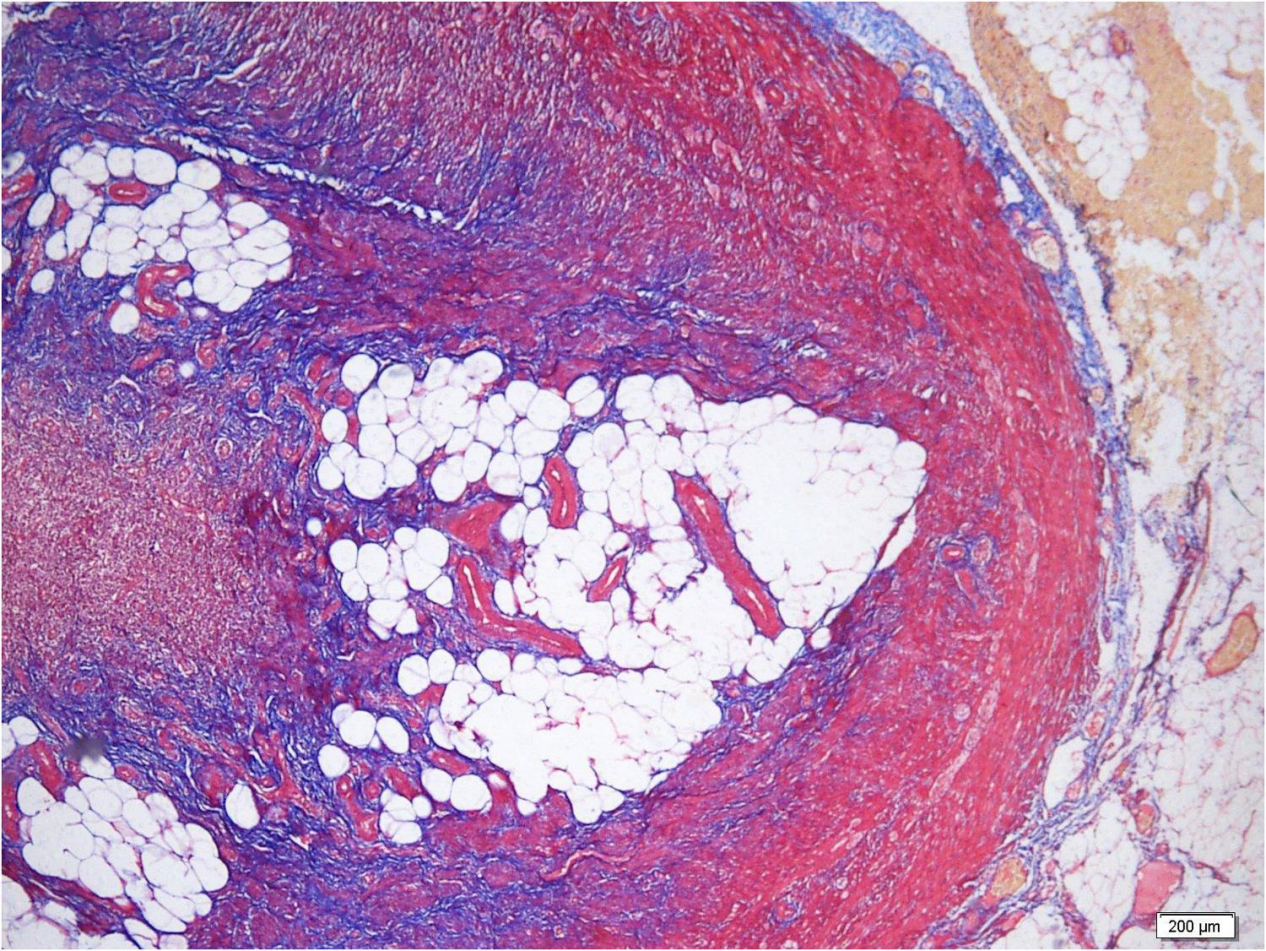
Advanced stromal and perineural fibrosis with luminal obliteration. Masson's trichrome staining highlighting dense collagen deposition and marked fibrotic remodeling (×4).

In the assessment of neural and stromal features, Masson's trichrome staining demonstrated stromal and perineural fibrosis in all re-evaluated specimens, with severity ranging from mild to severe and predominantly mild to moderate (Figure 1).

**Figure 2:**
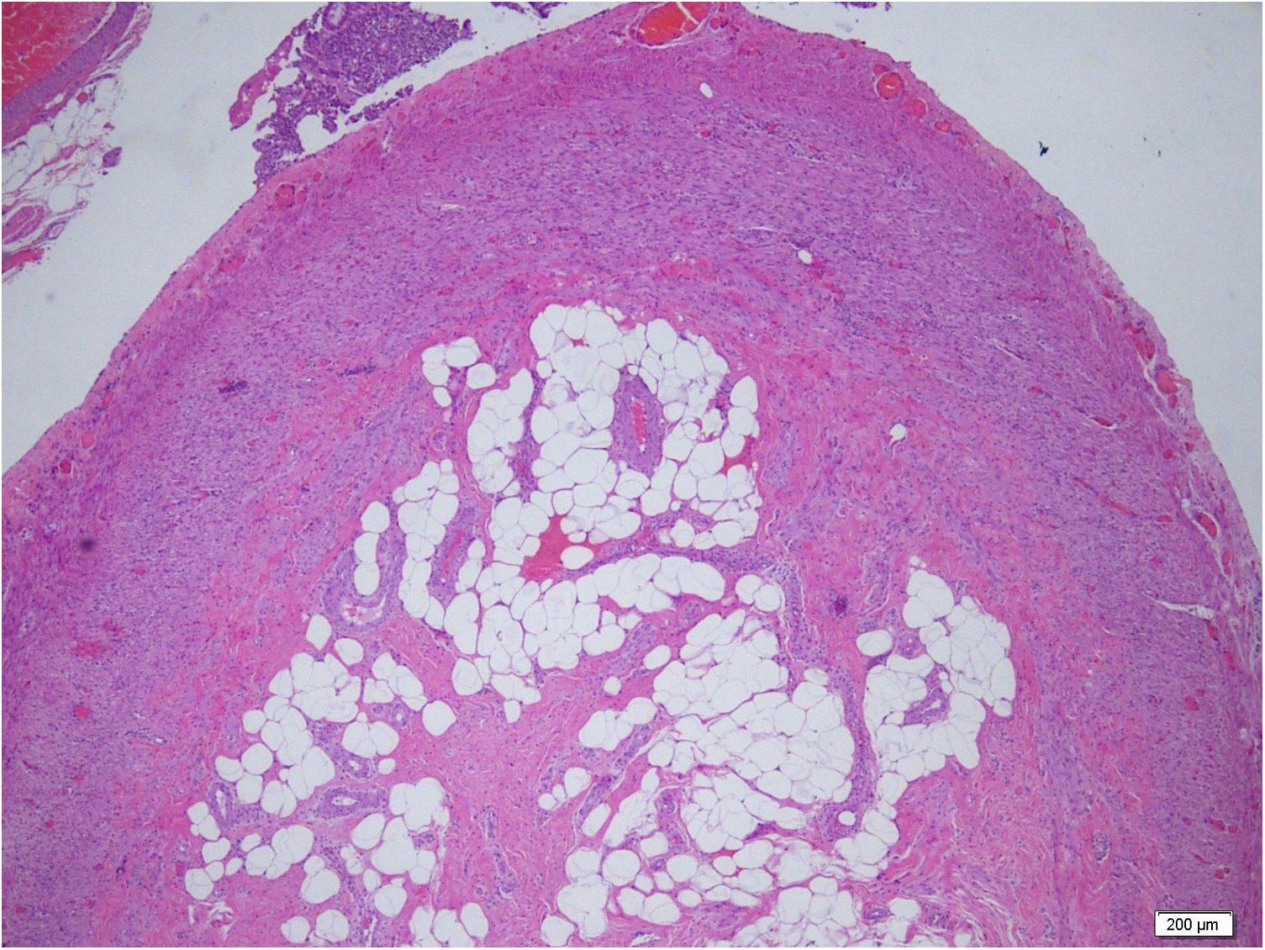
Complete fibrous obliteration of the appendiceal lumen. Hematoxylin and eosin staining demonstrating advanced fibrous replacement of the appendiceal lumen without evidence of acute inflammation. The image represents a single histological section (×4).

In a subset of specimens, partial or complete fibrous obliteration of the appendiceal lumen was identified on hematoxylin and eosin (H&E) sections (Figure 2).

**Figure 3:**
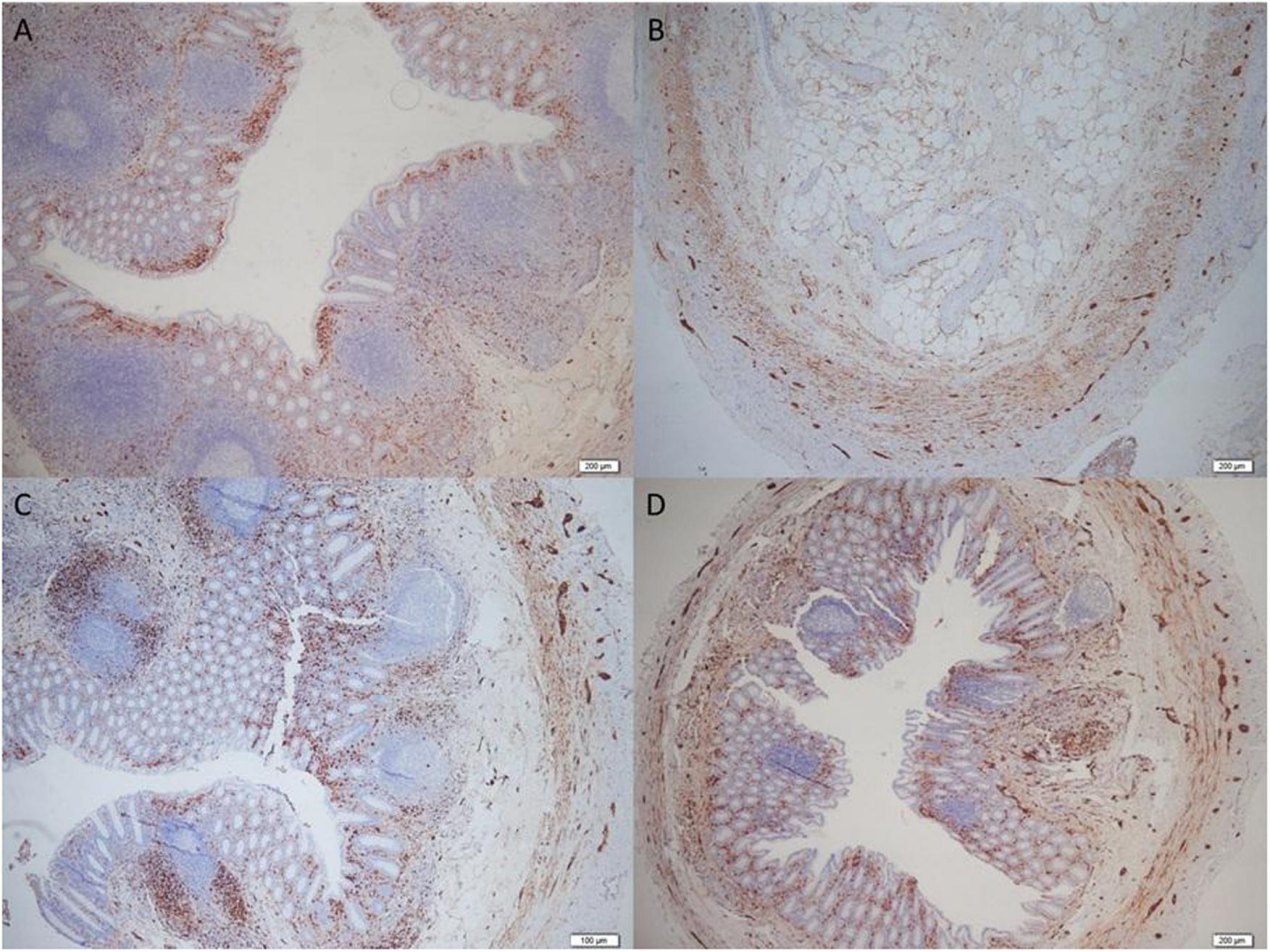
Höfler classification subtypes of reactive neural alterations in reactive lymphoid hyperplasia (RLH). Representative S-100–immunostained sections with high-magnification insets: **(A)** Type 1, mucosal; **(B)** Type 4, perineural fibrosis; **(C)** Type 3, submucosal neuromuscular proliferation; **(D)** Mixed (Type 1 + Type 3) patterns (×4; insets ×20).

**Figure 4:**
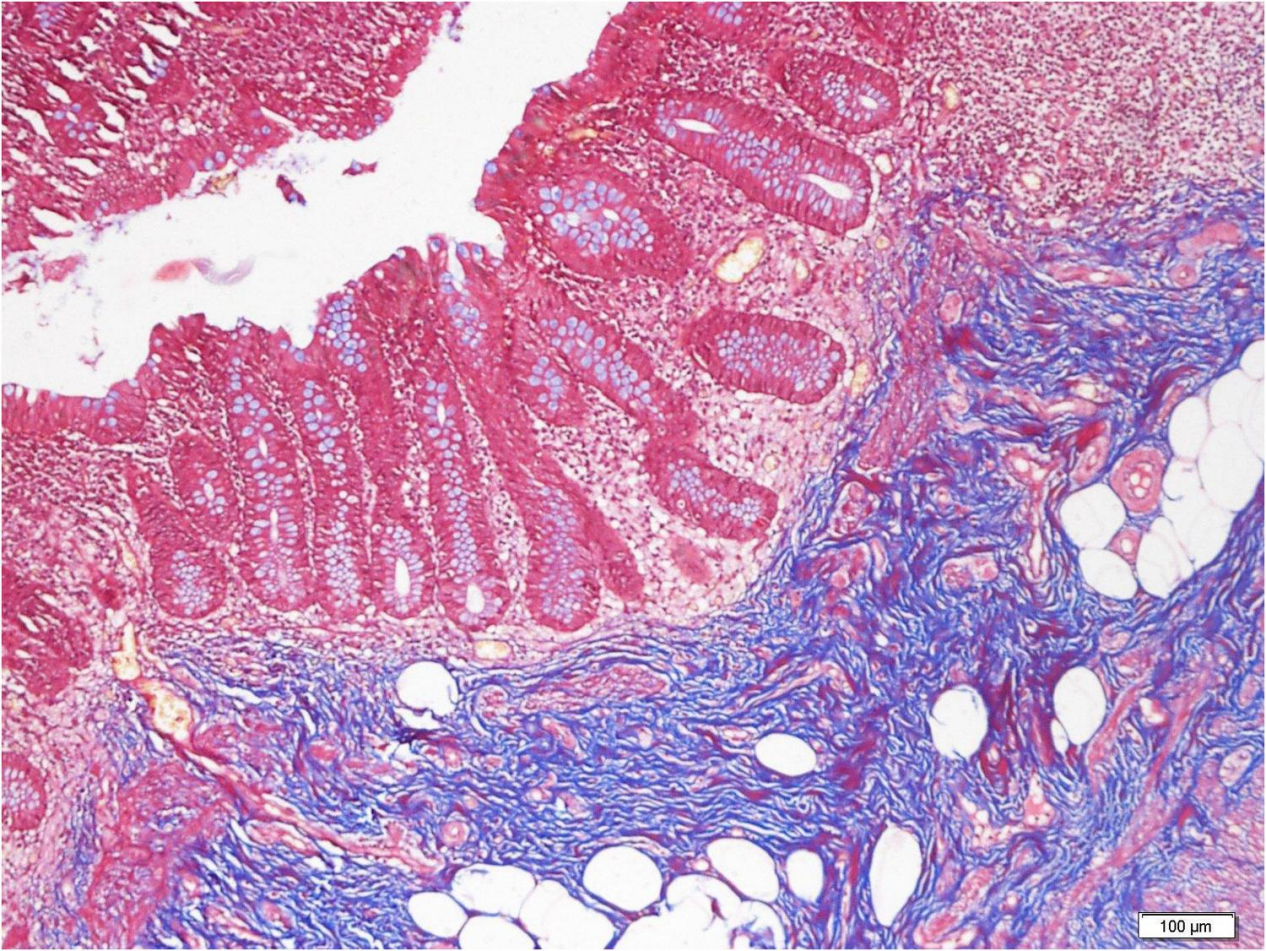
Stromal and submucosal fibrosis in RLH. Masson's trichrome staining demonstrating localized fibrotic expansion of the submucosal layer, including areas of perineural fibrosis, without neutrophilic inflammation (×10).

According to Höfler's classification (10), the distribution of neuropathic patterns was as follows. Type 1, the mucosal variant, was the most frequently observed pattern, identified in 46% of cases (Figure 3A). Type 4, perineural fibrosis, was also observed and is illustrated in Figure 3B, as well as in cases showing advanced stromal changes (Figure 4). Type 3, submucosal neuromuscular proliferation, was identified in a substantial subset of cases (Figure 3C). A mixed (Type 1 + Type 3) mucosal–submucosal pattern was observed in a small proportion of specimens (Figure 3D). Type 2, axial neuroma, was infrequently encountered (4% of cases) and is not illustrated in Figure 3.

### 3.3 Clinical outcome

Follow-up data were available for all patients, and symptom resolution was observed in all cases.

## 4 Discussion

This study constitutes a retrospective descriptive analysis of pediatric appendectomy specimens histopathologically diagnosed with RLH, focusing on associated clinical, imaging, and histopathological findings. RLH is frequently encountered in children presenting with abdominal pain; however, it continues to pose a diagnostic challenge because its clinical and radiological presentations may closely resemble those of acute appendicitis. In the absence of classical inflammatory changes, appendectomy specimens lacking an inflamed appendix are conventionally designated as “negative appendectomies” in surgical and radiological practice (4, 11, 14). However, this terminology does not fully capture the histopathological heterogeneity observed in these specimens and may oversimplify the underlying reactive processes.

In the present study, RLH specimens were systematically re-evaluated using S-100 immunohistochemistry and Masson's trichrome staining to characterize the accompanying neural and stromal features. Neuropathic alterations were classified according to Höfler's system, which delineates a spectrum of reactive neural patterns originally described in non-inflammatory appendiceal conditions. Importantly, the findings of this study demonstrate the presence of neural and stromal alterations in RLH specimens but do not establish that these changes are specific to RLH. The frequent identification of mucosal and submucosal neural elements, accompanied by varying degrees of perineural and stromal fibrosis, is consistent with the interpretation that these findings represent reactive or adaptive tissue responses rather than manifestations of a distinct neuropathic disease entity. Analogous neural proliferation has been documented across a broad spectrum of gastrointestinal conditions, including motility disorders, inflammatory bowel disease, and congenital anomalies (7–10, 12, 15, 16).

Neural proliferations within the appendix have been documented in pathology literature for many years. Masson first described mucosal nerve hyperplasia in appendiceal specimens, followed by observations by Maresch and subsequent classification by Höfler (9, 17, 18). Recent research has shown that neural proliferations may accompany fibrous obliteration of the appendiceal lumen, further supporting the concept that these alterations are nonspecific and can occur independently of acute inflammation (10). A recently described intramucosal variant of Schwann cell proliferation of the cecal appendix further highlights the histological diversity of appendiceal neural alterations and supports their characterization as reactive, nonspecific patterns (19).

In the present cohort, the identification of mucosal and submucosal neural elements alongside perineural fibrosis does not substantiate a causal relationship with symptom generation. These features are more plausibly attributable to secondary or adaptive tissue responses within an appendix characterized by prominent lymphoid hyperplasia. As an integral component of gut-associated lymphoid tissue, the appendix harbors a dense immune architecture and rich intrinsic innervation, which may render it susceptible to reactive neural changes in response to various immunological or luminal stimuli (3, 5, 19, 20). Consequently, although these findings add histopathological detail, they should not be interpreted as defining a novel clinicopathological entity.

It is well established that lymphoid hyperplasia may cause luminal obstruction of the appendix, thereby predisposing it to bacterial overgrowth and acute inflammation. In the present cohort, no specimen demonstrated histological evidence of luminal obstruction combined with secondary acute inflammatory changes that met the diagnostic criteria for acute appendicitis. Cases in which fibrous obliteration of the lumen was noted on H&E sections are documented in Table 2; however, these were not associated with transmural neutrophilic infiltration. The absence of obstructive-inflammatory appendicitis in this cohort should be noted.

Stromal and perineural fibrosis identified in RLH specimens is consistent with previously reported descriptions of reactive fibrotic alterations in the appendix (15, 21, 22). Fibrosis in the absence of acute inflammation is a nonspecific histopathological finding and may represent the cumulative effect of chronic or recurrent subclinical stimulation. Proposed mechanisms involving neuropeptide-mediated pathways or luminal obstruction remain speculative and cannot be substantiated solely by histological evidence. The relatively high frequency of neural and fibrotic alterations observed in this cohort should therefore be interpreted with caution.

In addition, methodological factors may have contributed to the increased detection of neural and stromal features. Additional tissue sampling, deeper sectioning, and the use of ancillary stains not routinely applied in standard pathological evaluation may have enhanced the visibility of these findings. Therefore, these observations may reflect increased detection sensitivity rather than true biological prevalence.

### 4.1 Limitations of the study

This study has several limitations that warrant consideration. First, in the absence of a histologically normal control group, it cannot be determined whether the observed neural and stromal alterations are specific to RLH, represent age-related variation, or reflect background histological features of the pediatric appendix. Published data indicate that S-100–positive neural elements and varying degrees of stromal fibrosis may also be present in normal or non-specifically inflamed appendices (12, 16). As appendectomy is rarely performed in asymptomatic children, and even incidentally resected appendices frequently demonstrate lymphoid hyperplasia (13), defining a true normal reference group remains inherently challenging.

Second, the retrospective design limited the ability to correlate histopathological findings with clinical symptoms, functional assessments, or long-term outcomes. Although postoperative symptom resolution was observed in patients with RLH, this does not imply a causal relationship between the identified neural or fibrotic features and preoperative symptomatology.

Third, generalizability may be influenced by geographic and epidemiological factors. In regions with a higher prevalence of gastrointestinal parasitic infections, appendiceal specimens may exhibit additional histological features. Although no parasitic infestation was identified in this cohort, systematic parasitological evaluation was not performed.

Notwithstanding these limitations, RLH accounted for 21.4% of all appendectomies in this cohort (100/468), consistent with previously reported pediatric rates (14, 23). Accurate histopathological characterization of these specimens is clinically relevant, facilitating communication between pathologists and clinicians and discouraging the reductive designation of “negative appendectomy.”

## 5 Conclusions

Reactive lymphoid hyperplasia (RLH) of the appendix is a common histopathological finding in children undergoing appendectomy for suspected acute appendicitis. In this descriptive study, RLH was associated with neural and stromal alterations, including mucosal nerve elements and perineural or stromal fibrosis. These findings should be interpreted cautiously and regarded as nonspecific reactive patterns rather than evidence of a distinct neuropathic entity.

Importantly, in the absence of a histologically normal control group, it cannot be determined whether these alterations are specific to RLH or represent background features of the pediatric appendix. In addition, the clinical significance of these findings remains uncertain, and no causal relationship can be inferred between the observed histopathological features and preoperative symptoms.

Although RLH does not fulfill the histological criteria for acute appendicitis, it should not be considered synonymous with a completely normal appendix. Careful histopathological documentation of RLH and its associated features may improve clinicopathological interpretation when inflammation is absent. Further prospective studies, ideally incorporating appropriate comparison groups and clinical correlation, are required to clarify the relevance, variability, and potential mechanisms of these reactive neural and stromal alterations.

## Data Availability

The original contributions presented in the study are included in the article/Supplementary Material, further inquiries can be directed to the corresponding author/s.
